# Supplementary material for: Tracking exceptional points above the lasing threshold
Source: Nat Commun. 2023 Dec 14;14:8304. doi: 10.1038/s41467-023-43874-z (PMC10721897; doi:10.1038/s41467-023-43874-z)
Supplement: Supplementary file 1 — Supplementary Information [file 41467_2023_43874_MOESM1_ESM.pdf]

Tracking exceptional points above the lasing threshold
Supplementary Information

Kaiwen Ji,[1] Qi Zhong,[2] Li Ge,[3,4] Gregoire Beaudoin,[1] Isabelle Sagnes,[1]
Fabrice Raineri,[1] Ramy El-Ganainy,[2,5,*] and Alejandro M. Yacomotti[1,6,†]

[1] *Centre de Nanosciences et de Nanotechnologies, CNRS, Université Paris-Saclay, 10 Boulevard Thomas Gobert, 91120 Palaiseau, France*
[2] *Department of Physics, Michigan Technological University, Houghton, Michigan 49931, USA*
[3] *Department of Physics and Astronomy, College of Staten Island, CUNY, Staten Island, New York 10314, USA*
[4] *Graduate Center, CUNY, New York, New York 10016, USA*
[5] *Henes Center for Quantum Phenomena, Michigan Technological University, Houghton, Michigan 49931, USA*
[6] *LP2N, Institut d'Optique Graduate School, CNRS, Université de Bordeaux, 33400 Talence, France*

Supplementary Note 1: Compensating the carrier-induced frequency detuning

As we mentioned in the main text, under asymmetric pumping of the two resonators, the carrier-induced frequency shift (which is proportional to the linewidth enhancement factor α) will break the parity symmetry between the two resonators. As a result, it is not possible to achieve PT symmetry and access the EP. In order to compensate for this dynamic effect, the two cavities must be designed to be initially asymmetric, i.e. having two different resonant frequencies. If the detuning is properly chosen, it can counterbalance the effect of the dynamic frequency shift. Here we derive the required frequency detuning to achieve this target. To do so, we start by considering the field equations of the laser rate model of Eq. (1a) in the main text, which can be expressed in the form:

$$-i\frac{d}{dt}\begin{bmatrix}a_1\\a_2\end{bmatrix} = H_{\mathrm{NL}}\begin{bmatrix}a_1\\a_2\end{bmatrix} \tag{S1}$$

where

$$H_{\mathrm{NL}} = \begin{bmatrix}(\omega_1+\Delta\omega_1)-ig_1 & K\\ K & (\omega_2+\Delta\omega_2)-ig_2\end{bmatrix},$$

$\Delta\omega_j = \frac{\alpha}{2}(n_j-n_0)\beta\gamma_{\|}$ and $g_j = -\kappa + (n_j-n_0)\beta\gamma_{\|}/2$, with $j=1,2$. Before we proceed, we emphasize the fact that the above equations incorporate the nonlinear effects arising from the coupling between the carrier and intensity. In other words, no linear approximations are made here. By expressing the fields as $a_j = A_j e^{i\phi_j} e^{i\Omega t}$ with A_j and ϕ_j being real numbers, we arrive at:

$$\Omega_\pm = \omega_{\mathrm{avg}} - ig_{\mathrm{avg}} \pm \sqrt{K^2 - \frac{(i\delta\omega_{12}+i\Delta\omega_{12}+\Delta g_{12})^2}{4}} \tag{S2}$$

where $\omega_{\mathrm{avg}} \equiv (\omega_1+\omega_2+\Delta\omega_1+\Delta\omega)/2$, $g_{\mathrm{avg}} \equiv (g_1+g_2)/2$, $\Delta\omega_{12} \equiv \Delta\omega_1 - \Delta\omega_2 = \frac{\alpha}{2}(n_1-n_2)\beta\gamma_{\|}$, $\Delta g_{12} \equiv g_1 - g_2$, and finally $\delta\omega_{12} \equiv \omega_1 - \omega_2$.

* ganainy@mtu.edu
† alejandro.giacomotti@c2n.upsaclay.fr

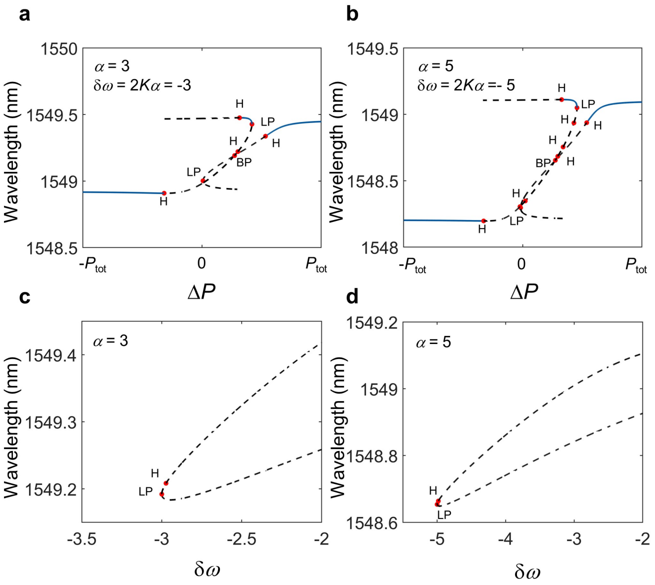

FIG. S1. (a)–(b) Compensation of nonlinearity for different α values. $K=0.5, P_{\mathrm{tot}} = 3P_0$. (c)–(d) Wavelength responses to the linear detuning at EP. Here $\Delta P|_{\mathrm{EP}}/P_0 = 0.82$.

In order to steer the system to an EP, the following two conditions must be satisfied: (i) $\delta\omega_{12} = -\Delta\omega_{12}$, (ii) $\Delta g_{12}/2 = K$. In writing this last relation, we assumed that $n_1 > n_2$ since here $K > 0$. Obviously, the other choice could have been made as well. Taken together, these two conditions lead to

$$\delta\omega_{12} = -2K\alpha. \tag{S3}$$

Importantly, since the lasing frequencies $\Omega_\pm$ must be real, we find that, at the EP —and this even for $K > \Delta g_{12}$—, $g_{\mathrm{avg}} = 0$, or equivalently $g_1 = -g_2$. In other words, at the EP, the system respects PT symmetry.

In order to confirm the validity of this analysis, we have calculated the lasing modes of two asymmetric laser cavities under the above derived detuning conditions. Figure S1(a) and (b) display the resulting bifurcation diagrams for different values of α and the corresponding detuning. For both cases, effective PT symmetry is resorted at

the branch points, which suggests that the compensation scheme described above can work under a wide range of parameters. Unlike a pitchfork, the bifurcation at the EP as ΔP is changed is a *transcritical* one: locally, there are two fixed points before and after the branch point. Importantly, while the frequency scaling is linear in the vicinity of the EP, it becomes square-root as the bifurcation parameter is the detuning [Figs. S1(c) anb (d)].

Supplementary Note 2: Locating the EP in the parameter space

In this note, we derive the condition for operating at an EP above the lasing threshold. To do so, we start by recalling the characteristics of a lasing EP, which can be summarized as follows: (i) $\delta\omega = -2K\alpha$, (ii) $g_1 = -g_2$, (iii) The fields in both cavities have equal amplitude, and (iv) The phases of the fields in both cavities differ by $\pi/2$. As discussed before, the first of these conditions is the frequency detuning required to compensate for the carrier-induced frequency shift at the EP. When this condition is satisfied, the second relation becomes the lasing condition (imaginary part of $\Omega_\pm = 0$) at the EP. The third and fourth arise directly from the expression for the exceptional eigenvector associated with the solution of the matrix eigenvalue problem in Eq.(S1). In other words, we have:

$$\frac{1}{2}(n_1 - n_0)\beta\gamma_\parallel - \kappa = -\left[\frac{1}{2}(n_2 - n_0)\beta\gamma_\parallel - \kappa\right] \quad \text{(S4)}$$

$$\frac{\alpha}{2}(n_1 - n_0)\beta\gamma_\parallel = \frac{\alpha}{2}(n_2 - n_0)\beta\gamma_\parallel + 2K\alpha \quad \text{(S5)}$$

$$|a_1|^2 = |a_2|^2, \quad \text{(S6)}$$

$$\phi_1 - \phi_2 = \pi/2. \quad \text{(S7)}$$

By solving Eqs. (S4) and (S5), we can obtain the following expressions for the carrier numbers at EP:

$$n_1 = n_0 + \frac{2(K+\kappa)}{\beta\gamma_\parallel}, \quad \text{(S8)}$$

$$n_2 = n_0 - \frac{2(K-\kappa)}{\beta\gamma_\parallel}. \quad \text{(S9)}$$

Meanwhile, these carrier numbers can be related to the pump rates by solving the carrier rate equations [Eq.(1b) in the main text] under steady states conditions, i.e., $\dot{n_1} = \dot{n_2} = 0$. By doing so, we arrive at:

$$P_j - n_j\gamma_{\text{tot}} = (n_j - n_0)\beta\gamma_\parallel |a_j|^2. \quad \text{(S10)}$$

From Eq. (S6) and Eq. (S10) we obtain:

$$\frac{n_1 - n_0}{n_2 - n_0} = \frac{P_1 - \gamma_{\text{tot}} n_1}{P_2 - \gamma_{\text{tot}} n_2}. \quad \text{(S11)}$$

Finally, by combining Eqs. (S9), (S10) and (S11), we obtain:

$$\Delta P|_{\text{EP}} = \frac{K(P_{\text{tot}} - 2n_0\gamma_{\text{tot}})}{\kappa}, \quad \text{(S12)}$$

where $\Delta P = P_1 - P_2$. Recalling the definition of the gain difference at EP $\Delta g_{12}|_{\text{EP}} = \beta\gamma_\parallel(n_1 - n_2)/2 = 2K$, we can rewrite Eq. (S12) as

$$\Delta P|_{\text{EP}} = \frac{\Delta g_{12}|_{\text{EP}}(P_{\text{tot}} - 2n_0\gamma_{\text{tot}})}{2\kappa}, \quad \text{(S13)}$$

which shows that the pump difference at the exceptional point, $\Delta P|_{\text{EP}}$, is not only a function of the gain difference, but also of the total pump power. At the laser threshold, it takes the simple form $\Delta P|_{\text{EP}}^{\text{th}} = (P_1 - P_2)|_{\text{EP}}^{\text{th}} = 2\Delta g_{12}|_{\text{EP}}\gamma_{\text{tot}}/\beta\gamma_\parallel$, which recovers the linear case.

On the other hand, in the high pumping limit Eq. (S12) reduces to

$$\lim_{P_{\text{tot}}\to\infty} \frac{\Delta P|_{\text{EP}}}{P_{\text{tot}}} = \frac{K}{\kappa}, \quad \text{(S14)}$$

which implies that the system parameters must satisfy the condition $K < \kappa$, meaning that the coupling between the two cavities must be smaller than the loss factor of each cavity (weak intercavity coupling conditions).

Finally, in the general case, Eq. (S9) gives an upper bound for the coupling strength leading to an EP above the laser threshold: $K \leq K_{\max} \equiv \kappa + \frac{1}{2}n_0\beta\gamma_\parallel$. The limit value $K_{\max}$ corresponds to the EP at laser threshold ($|a_1|^2 = |a_2|^2 = 0$) when only one of the cavities is pumped, say $P_1 = P_{\text{tot}} = P_{\text{EP}}^{\text{th}}$ and $P_2 = 0$. For the parameters used in our work (see note for a full list of parameters), this becomes $K_{\max}/\kappa = 3.12$.

Supplementary Note 3: Lasing threshold, intensity and frequency at the EP

At threshold, we have $|a_1|^2 = |a_2|^2 = 0$. From Eq. (S10), we thus obtain:

$$P_j^{\text{th}} = n_j\gamma_{\text{tot}}. \quad \text{(S15)}$$

By using Eqs. (S8) and (S9), find:

$$P_{\text{EP}}^{\text{th}} = P_1^{\text{th}} + P_2^{\text{th}} = 2\left(n_0 + \frac{2\kappa}{\beta\gamma_\parallel}\right)\gamma_{\text{tot}} = 2P_0, \quad \text{(S16)}$$

where P_0 is the threshold of the single cavity laser. This last relation confirms that $P_{\text{tot}} > 2n_0\gamma_{\text{tot}}$ above the lasing threshold. We have also numerically evaluated the lasing threshold in the broken and unbroken PT phases, namely, $\Delta P = P_{\text{tot}}$ and $\Delta P = -P_{\text{tot}}$, respectively, as functions of the coupling coefficient K. This result is shown in Fig. S2(a).

The intracavity intensity at the EP reads

$$I_{\text{EP}} = \frac{P_{\text{tot}} - 2n_0\gamma_{\text{tot}}}{4\kappa} - \frac{\gamma_{\text{tot}}}{\beta\gamma_\parallel}. \quad \text{(S17)}$$

Finally, in order to determine the lasing frequency at EP, we recall that at or above threshold $g_{\text{avg}} = 0$. Moreover,

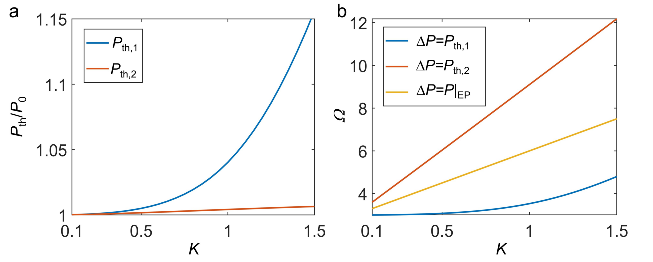

FIG. S2. (a) Threshold of the single cavity pumped case, the blue and red curves represent the threshold for pump cavity 1 and cavity 2, respectively. (b) Lasing frequency of the single-cavity pumped case (blue and red curves) and the frequency at EP (yellow curve).

at EP, $\Omega_\pm = \omega_{\rm avg}$. By recalling that $\omega_2 = \omega_1 + 2K\alpha$ and that the lasing condition $g_1 = -g_2$ implies that $\sum_{j=1}^{2}(n_j - n_0)\beta\gamma_{\|} = 2\kappa$, we find:

$$\Omega_{\rm EP} = \omega_1 + \alpha(\kappa + K). \tag{S18}$$

In Fig. S2 (right panel) we display the laser frequencies in the three following cases for comparison: the frequency at the EP [yellow curve, Eq. (S18)] and the frequencies when a single cavity is pumped at its threshold ($\Delta P = P_{\rm th,1}, \Delta P = -P_{\rm th,2}$, blue and red curves, respectively). We observe, as it appears in the bifurcation diagrams in the main text —and also in Figs. S1(a)-(b)— that the single cavity mode ($\Delta P = P_{\rm th,1}$) is red-shifted compared to the EP, while it is blue-shifted for another case ($\Delta P = -P_{\rm th,2}$).

Supplementary Note 4: Light-in Light-out curve

Figure S3 shows the light-out vs light-it curve for the unbalanced pump configuration. The model predicts that the blue-detuned cavity (cavity 2) has a lower threshold compared with cavity 1, $P_{\rm th,2} < P_{\rm th,1}$ [Fig. S1 (a)]. Such a difference is expected to be weak in the weak coupling regime ($K < \kappa$), *e.g.* $(P_{\rm th,2} - P_{\rm th,1})/P_0 \approx 0.03$ for $K/\kappa = 0.95$. Experimentally, though, we observe $P_{\rm th,1} - P_{\rm th,2} \sim 15\%$, which might be attributted to slight differences in the alignment of the SLM-shaped pump spots, and/or slightly different cavity Q-factors.

Supplementary Note 5: Stability of the solutions

To calculate lasing modes and their bifurcation diagrams, we express the complex fields as $a_j = A_j(t)e^{i\omega t + i\phi_j(t)}$. By substituting back in the laser rate

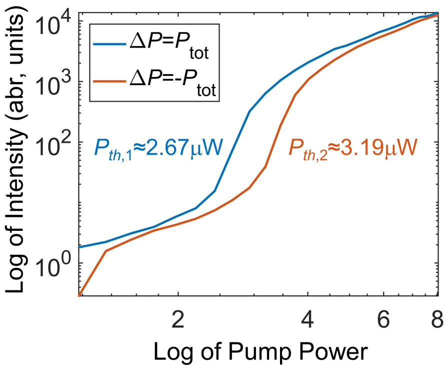

FIG. S3. Experimental measurement of the lasing threshold of the system when a single cavity is pumped. The blue curve stands for the threshold for pumping cavity 1, and the red one is the threshold curve for pumping cavity 2.

equations (Eq.(1a) in the main text) we obtain:

$$\dot{A}_1 = \frac{1}{2}\left[\beta\gamma_{\|}(n_1 - n_0) - 2\kappa\right] A_1 + KA_2 \sin\Delta\phi, \tag{S19}$$

$$\dot{A}_2 = \frac{1}{2}\left[\beta\gamma_{\|}(n_2 - n_0) - 2\kappa\right] A_2 - KA_1 \sin\Delta\phi, \tag{S20}$$

$$\dot{\Delta\phi} \equiv \dot{\phi}_1 - \dot{\phi}_2 = \frac{1}{2}\alpha\beta\gamma_{\|}(n_1 - n_0) + \frac{A_2}{A_1}K\cos\Delta\phi - \left[\frac{1}{2}\alpha\beta\gamma_{\|}(n_2 - n_0) + \frac{A_1}{A_2}K\cos\Delta\phi\right] + \delta\omega. \tag{S21}$$

The lasing modes and their bifurcation diagrams are then calculated by solving these equations together with the carrier rate equations [Eq.(1b) in the main text] by using the open source continuation software package MatCont [1]. Accessing a particular mode using numerical integration of the laser equations can be achieved by choosing the proper initial noise in the system.

The stability of the modes is calculated by using linear stability analysis around the lasing modes, i.e. using the Jacobian matrix:

$$\boldsymbol{J} = \begin{bmatrix} \frac{\partial f_{A_1}}{\partial A_1} & \frac{\partial f_{A_1}}{\partial A_2} & \frac{\partial f_{A_1}}{\partial n_1} & \frac{\partial f_{A_1}}{\partial n_2} & \frac{\partial f_{A_1}}{\partial \Delta\phi} \\ \frac{\partial f_{A_2}}{\partial A_1} & \frac{\partial f_{A_2}}{\partial A_2} & \frac{\partial f_{A_2}}{\partial n_1} & \frac{\partial f_{A_2}}{\partial n_2} & \frac{\partial f_{A_2}}{\partial \Delta\phi} \\ \frac{\partial f_{n_1}}{\partial A_1} & \frac{\partial f_{n_1}}{\partial A_2} & \frac{\partial f_{n_1}}{\partial n_1} & \frac{\partial f_{n_1}}{\partial n_2} & \frac{\partial f_{n_1}}{\partial \Delta\phi} \\ \frac{\partial f_{n_2}}{\partial A_1} & \frac{\partial f_{n_2}}{\partial A_2} & \frac{\partial f_{n_2}}{\partial n_1} & \frac{\partial f_{n_2}}{\partial n_2} & \frac{\partial f_{n_2}}{\partial \Delta\phi} \\ \frac{\partial f_{\Delta\phi}}{\partial A_1} & \frac{\partial f_{\Delta\phi}}{\partial A_2} & \frac{\partial f_{\Delta\phi}}{\partial n_1} & \frac{\partial f_{\Delta\phi}}{\partial n_2} & \frac{\partial f_{\Delta\phi}}{\partial \Delta\phi} \end{bmatrix} \tag{S22}$$

where f_ξ defined as $\dot{\xi} = f_\xi(A_1, A_2, n_1, n_2, \Delta\phi)$ (for $\xi = A_j, n_j, \Delta\phi$), represent the fixed points of the nonlinear dynamical equations, namely $\dot{\xi} = 0, \dot{n_j} = 0, \dot{\Delta\phi} = 0$. Negative and positive values of the real part of the complex eigenvalues of the Jacobian then indicate stable and unstable solutions, respectively.

Interestingly, the bifurcation diagrams in both the main text and the Supplementary Information show that

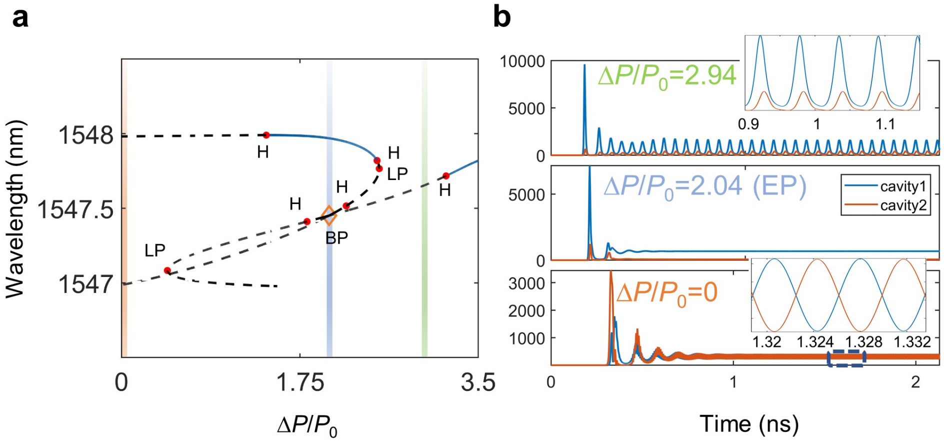

FIG. S4. **a** Bifurcation diagram and stability calculated for $P_{\rm tot} = 3.5P_0$. **b** Temporal dynamics for a Q-switch like pulsation (top panel), an exemplary stable solution (middle panel) and one unstable, oscillator, solution (bottom panel)
.

there exists a regime around the bifurcation point where all the fixed points are unstable. As illustrated in Fig. S4, which is calculated for $P_{\rm tot} = 3.5P_0$, the system does not admit stable steady state solutions but rather oscillates in time. Two important non-steady-state scenarios can be identified in Fig. S4(b): perfect pump balance ($\Delta P = 0$, bottom panel), showing a mode beating limit cycle, and Q-switch self-pulsing, whose oscillation time-scale is much slower and scales with the carrier-recombination lifetime ($\Delta P = 2.94P_0$, top panel). For pump unbalance at the EP the system eventually relaxes to the only stable branch solution [blue solid line in Fig. S4(a), $\Delta P = 2.04P_0$].

Supplementary Note 6: Stochastic analysis of laser rate equations

In the main text, we presented a comparison between the experimental results and numerical simulations obtained by solving the laser rate equations in the presence of stochastic noise. Among the different algorithms for integrating stochastic differential equations, here we employed the Eurler-Maruyama method [2]. The noise terms, which arise due to spontaneous emission, were taken to be a white noise: $\langle F_\mu(t)F_\nu(t')\rangle = 2D_{\mu\nu}\delta(t-t')$. The coefficient is $2D_{a_ia_i^*} = 2D_{a_ia_i^*} = R_{\rm SP}$, with $R_{\rm SP}$ being the spontaneous emission rate $R_{\rm SP} = \beta F_p B n_{1,2}^2/V_a$, where F_p is Purcell factor, B is the bimolecular radiative recombination rate and V_a is the volume of the active medium [3]. The spectra are obtained after the Fourier transformation of the numerical integration. The numerical values of parameters used in the simulations are listed in table S1.

TABLE S1. Parameter values

Symbol	Values
κ	140.86GHz
α	3
β	0.017
$\gamma_{\|\|}$	2.2GHz
$\gamma_{\rm tot}$	5GHz
V_a	0.016×10^{-12}cm^3
n_0	10^{18} cm$^{-3}V_a = 16000$
F_p	1.03
B	$3{\times}10^{10}$ cm^{3}s^{-1}

Supplementary Data

For completeness, in this section, we present more measurement data for the lasing mode wavelengths as a function of $\Delta P \in [-P_{\rm tot}, P_{\rm tot}]$ evaluated at different values of $P_{\rm tot}$. These results are plotted in Fig. S5. By tracking the location of the bifurcation point in each figure, it is clear that the trend discussed in the main text (i.e. the shift of the bifurcation point toward larger positive values of ΔP as $P_{\rm tot}$ increased does persist. On the other hand, Fig. S6 depicts the experimental (left panel) and numerical (right panel) results for the lasing characteristics of this system in the absence of frequency detuning compensation. In this case, there is no EP in the system and the lasing characteristics are considerably less sensitive to the total pump power.

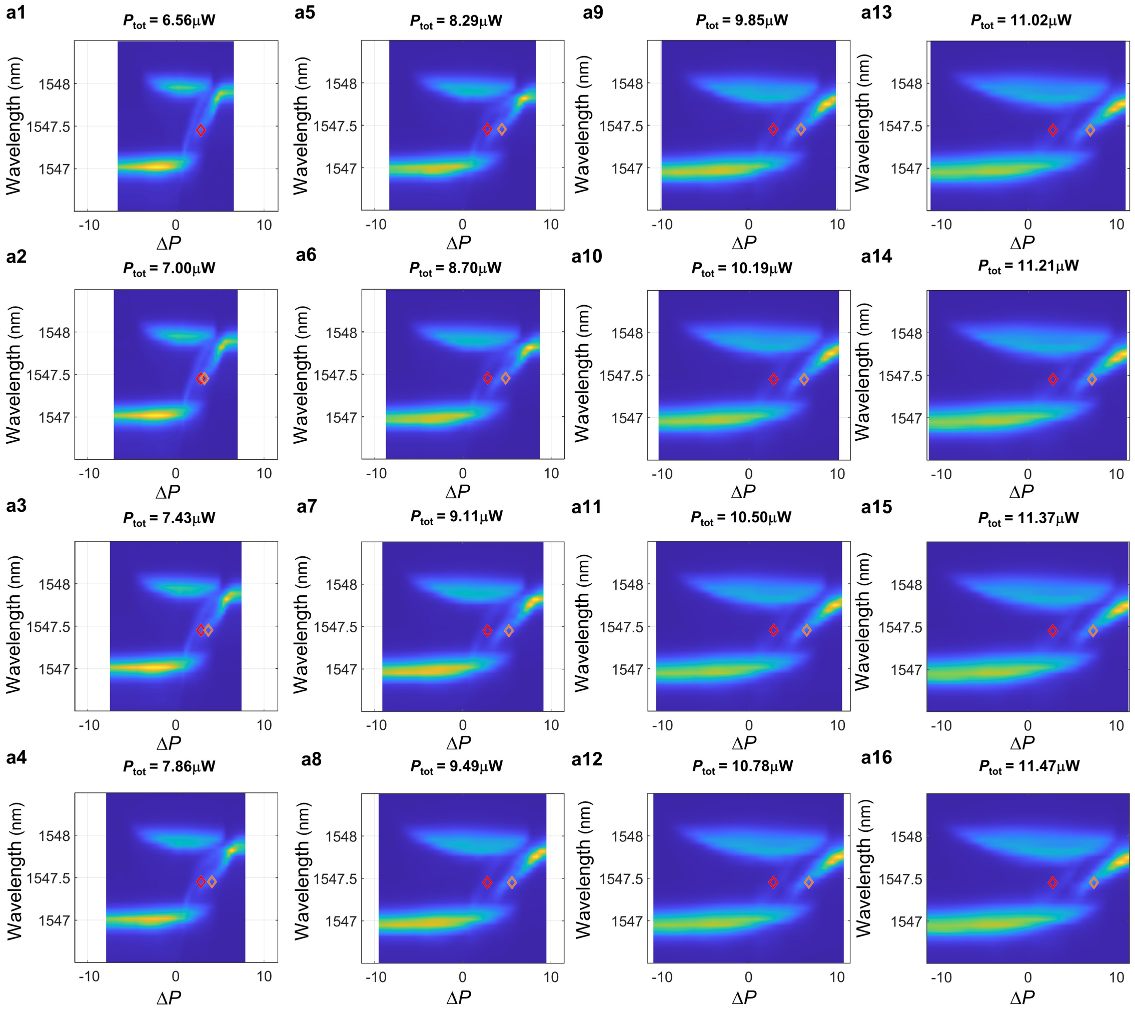

FIG. S5. Extended data for the measurements of the lasing modes as a function of ΔP for different values of P_{tot}. The same trend discussed in the main text is observed here as well, which supports the conclusion of this work.

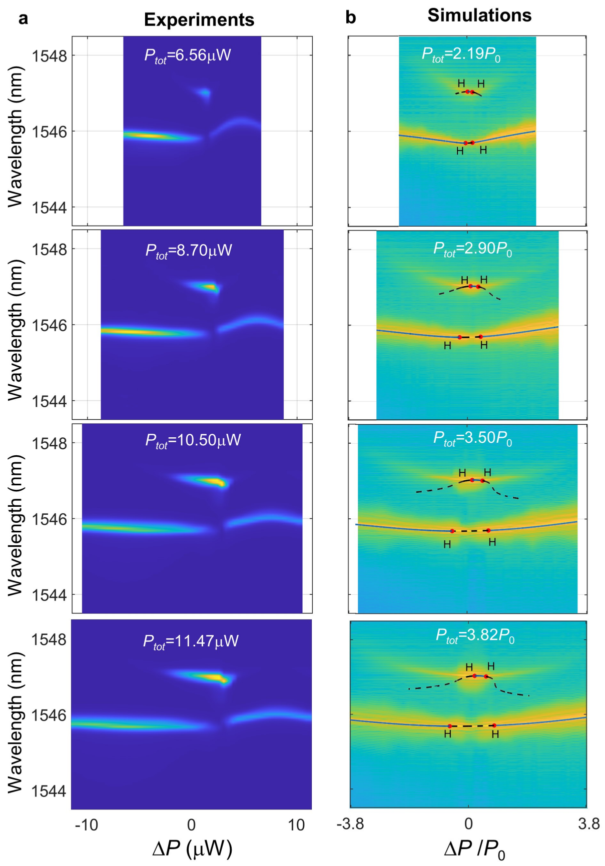

FIG. S6. Similar measurements to those presented in Fig.3 in the main text but for a different sample having $\delta\omega = 1.19 \ll 2K\alpha = 22.38$ ($K = 3.73$), i.e. with no frequency compensation to counterbalance the carrier-induced blueshift. As before, left panels present experimental data while right panels depict theoretical results. In contrast to the behavior observed in Fig. 3 in the main text, here the lasing spectral pattern remains almost invariant as the total pump power is varied. Panel (a) display the experimental results and the corresponding simulations are shown in (b).

Supplementary References

[1] A. Dhooge, W. Govaerts, Y. A. Kuznetsov, H. G. E. Meijer, and B. Sautois, Mathematical and Computer Modelling of Dynamical Systems **14**, 147 (2008).

[2] P. E. Kloeden and E. Platen, in *Numerical solution of stochastic differential equations* (Springer, 1992) pp. 103–160.

[3] M. Marconi, F. Raineri, A. Levenson, A. M. Yacomotti, J. Javaloyes, S. H. Pan, A. El Amili, and Y. Fainman, Phys. Rev. Lett. **124**, 213602 (2020).
